# Glutamine in critically ill patients: is it a fundamental nutritional supplement?

**DOI:** 10.5935/0103-507X.20160022

**Published:** 2016

**Authors:** Paulo Martins

**Affiliations:** 1 Serviço de Medicina Intensiva, Centro Hospitalar Universitário de Coimbra - Coimbra, Portugal.

Glutamine is the most abundant free amino acid in the human body and is necessary to modulate the inflammatory and oxidative stress responses in patients.^(1,2)^

Systemic glutamine availability is determined by the balance of endogenous glutamine production (mainly in muscular tissue) and its use by glutamine consuming organs (gut, kidney, liver and the immune system).

Several studies show that in catabolic intensive care unit (ICU) patients, the endogenous production of muscular glutamine is increased while the plasma levels of glutamine are decreased, indicating elevated glutamine needs.^(3,4)^ We know also that low plasma glutamine values (< 420µmol/L) upon admission are related to increased mortality.^(5)^ These findings are the rationale for the use of glutamine supplementation in the ICU population in order to replenish the muscle pool of glutamine, attenuate the efflux of this amino acid, and provide exogenous glutamine required to meet the elevated organ needs for improvement in protein synthesis, modulation of the immune system, reduction of oxidative stress, and preservation of the gut barrier.

However, recent observations challenge this hypothesis. The plasma levels of glutamine are extremely variable in the ICU population^(6)^ and are not always associated with increased mortality.^(7)^ The glutamine supplementation does not stop the glutamine efflux from muscle because the endogenous muscular production and plasma levels of glutamine are related to the severity of the illness.^(8)^

Intensive care unit patients are considered to be immunosuppressed as evidenced by reduced levels of and the presence of dysfunctional T lymphocytes, altered neutrophil activity and an imbalance in the production of cytokines.^(9-13)^

The demonstration of the benefit of glutamine in increasing the number and functionality of effector cells of the immune response is evident in some studies,^(14-19)^ while in others that answer is not so obvious.^(20,21)^ We found (personal communication)^(22)^ that in the ICU population, glutamine supplementation (0.40g/Kg/day) via parenteral nutrition improved cellular immune function with significant increases in CD14 and CD14 HLA-DR monocytes and significant decreases in T regulatory cells with a significant reduction in the appearance of nosocomial infection.

Over the years, many studies examining glutamine in ICU populations have presented controversial results. We can differentiate between the small monocentric studies of the early years showing a significant reduction in mortality and infectious morbidity with glutamine supplemented mainly by parenteral nutrition from the more recent large, multicenter studies in which the benefits of reducing infectious morbidity and mortality observed in the early studies are lost.^(23)^

Among the various trials, we chose to highlight two large, multicenter, randomized studies, the REDOX study and the Signet trial, because they put into question the safety and efficacy of the use of glutamine in critically ill patients.

The REDOX study^(24)^ is a well conducted, large, multicenter trial illustrating the fact that early administration of glutamine in high doses (much higher than recommended) can have adverse effects. This was reflected by increased mortality in the group supplemented with glutamine in patients with multi-organ failure (including kidney dysfunction), some of whom had high basal serum levels of glutamine. Some authors had already reported that elevated serum levels of glutamine have been associated with higher mortality.^(7)^

The Signet study^(25)^ provided evidence that parenteral nutrition supplementation with low doses (20,2g/day) of glutamine for short periods of time did not influence the mortality or the infection incidence in the ICU population.

Several published meta-analyses were influenced by the progress of studies over time,^(23,26-29)^ which initially showed a significant reduction in mortality and infectious morbidity.^(18,30-32)^ Now, with the inclusion of recent studies, no effect of glutamine supplementation on mortality and a slight tendency toward reduction of infectious morbidity has been observed.^(24,25,33-35)^ The large multicenter studies mentioned before significantly contributed to the final results of the recent meta-analyses because of their impact.

It is not easy to obtain a clear answer to the above quoted question, as the critically ill population is a heterogeneous one. Studies often mix patients with different pathologies and prognoses, as well as include distinct routes of administration and the use of different doses than those recommended by the guidelines, thus giving mixed results, especially when compared to a meta-analysis.

The effects of parenteral glutamine supplementation on mortality differed with patient population, modes of nutrition and glutamine dosages. Taking into account these assumptions, glutamine supplementation confers no additional benefit in reducing mortality among the critically ill population. There are differences in sub-populations of ICU patients with a beneficial improvement in the surgical population versus medical or mixed ICU populations. Parenteral supplemented glutamine reduces nosocomial infections in critically ill patients.^(29,36)^

Available data on glutamine supplemented via parenteral nutrition cannot be compared to enteral nutritional supplementation and therefore cannot be used as the basis for recommendation of enteral glutamine administration.

Enteral glutamine supplementation does not confer significant benefits in the treatment of critically ill patients. In burned patients, there may be a benefit in reducing mortality and infectious morbidity, but data are scarce, and broader studies are warranted to confirm this effect.^(37,38)^

Meta-analyses have to be regarded with the necessary reservations; results should not be taken as dogma but rather, as sources to generate hypotheses for future studies.

Despite this apparent paradigm change, there is sufficient evidence in the literature on the benefits of glutamine that impel us to continue research by putting forth new questions.

We do not share the view that low glutamine plasma levels might be considered an adaptive response, which when disturbed by supplemented glutamine may induce deleterious effects.^(39)^ The beneficial effects of glutamine on immunological regulation of immunodepressive conditions and the reduction in nosocomial infections contradict this theory.

Glutamine is required for multiple metabolic pathways in maintaining the structure and function of various organ cells, so we must learn the lessons of the past and reformulate new studies, trying to determine if glutamine remains useful as a nutritional supplement for critically ill patients.

## What should the researchers of glutamine effects on the intensive care unit population be aware of in the future?

In future studies, we need to learn more about glutamine kinetics, the relationship between plasma glutamine concentration, and endogenous glutamine production along the evolution of critically ill patients. It will be important to know whether glutamine kinetics always have the same pattern or if it varies with the pathological situation in order to determine to whom and when to supplement glutamine.

Future research must explore the mechanism by which a glutamine deficiency could be harmful for some patients.

The researchers must put forth the right questions, such as the following:

Are all patients in the ICU candidates for therapy with glutamine, or are only the ICU patients with glutamine deficiency appropriate for therapy? What is the right dose of glutamine supplementation? Do all patients of the heterogeneous ICU population have the same needs, or are there specific needs for specific sub-populations?

There are still too many questions in the air, so the glutamine story will continue...
